# Correction: Williams et al. Kinetics and Kinematics of Working Trials Dogs: The Impact of Long Jump Length on Peak Vertical Landing Force and Joint Angulation. *Animals* 2021, *11,* 2804

**DOI:** 10.3390/ani12020134

**Published:** 2022-01-07

**Authors:** Ellen Williams, Anne Carter, Jacqueline Boyd

**Affiliations:** 1 Department of Animal Health, Behaviour & Welfare, Harper Adams University, Shropshire TF10 8NB, UK; ewilliams@harper-adams.ac.uk; 2 School of Animal, Rural & Environmental Sciences, Nottingham Trent University, Nottingham NG25 0QF, UK; Jacqueline.boyd@ntu.ac.uk

The authors wish to make the following correction to this paper [1]:

**Figure Legends**

In the original article, there was a mistake in the legends for Figure 4, Figure 5 and Figure 6. The correct legends appear below. The authors apologize for any inconvenience caused and state that the scientific conclusions are unaffected. The original article has been updated.

**Page 7, the legend of Figure 4:** “N” needs to be replaced by “lbs”; the correct version is as follows:

Maximum peak vertical landing force (lbs) for the study dogs (*n* = 21) at the three long jump lengths (7 ft, 8 ft, 9 ft). 

**Page 7, the legend of Figure 5:** “N/kg” needs to be replaced by “lbs/kg”; the correct version is as follows:

Peak vertical landing force (PVF) as a proportion of body weight (lbs/kg) across all the study dogs (*n* = 21) at the three long jump lengths (7 ft, 8 ft, 9 ft). 

**Page 9, the legend of Figure 6:** “N” needs to be replaced by “lbs”; the correct version is as follows:

Difference in peak vertical landing force (lbs) between the two front feet for the study dogs (*n* = 21) at the three long jump lengths (7 ft, 8 ft, 9 ft). 

The authors would like to apologize for any inconvenience to the readers caused by these errors.

## References

[1] Williams E., Carter A., Boyd J. (2021). Kinetics and Kinematics of Working Trials Dogs: The Impact of Long Jump Length on Peak Vertical Landing Force and Joint Angulation. Animals.

